# Determination of Zinc, Cadmium and Lead Bioavailability in Contaminated Soils at the Single-Cell Level by a Combination of Whole-Cell Biosensors and Flow Cytometry

**DOI:** 10.3390/s150408981

**Published:** 2015-04-16

**Authors:** Quentin Hurdebise, Cédric Tarayre, Christophe Fischer, Gilles Colinet, Serge Hiligsmann, Frank Delvigne

**Affiliations:** 1 Bât. G1 Bio-Industries, Passage des Déportés 2, Gembloux Agro-Bio Tech, University of Liège, 5030 Gembloux, Belgium; E-Mails: quentin.hurdebise@ulg.ac.be (Q.H.); cedric.tarayre@ulg.ac.be (C.T.); christophe.fischer@ulg.ac.be (C.F.); s.hiligsmann@ulg.ac.be (S.H.); 2 Bât. G1 Systèmes Sol-Eau, Avenue Maréchal Juin 27, Gembloux Agro-Bio Tech, University of Liège, 5030 Gembloux, Belgium; E-Mail: Gilles.Colinet@ulg.ac.be

**Keywords:** flow cytometry, *Escherichia coli* pP_ZntA_gfp, cadmium, lead, zinc, bioavailability, whole-cell biosensors

## Abstract

Zinc, lead and cadmium are metallic trace elements (MTEs) that are widespread in the environment and tend to accumulate in soils because of their low mobility and non-degradability. The purpose of this work is to evaluate the applicability of biosensors as tools able to provide data about the bioavailability of such MTEs in contaminated soils. Here, we tested the genetically-engineered strain *Escherichia coli* pP_ZntA_gfp as a biosensor applicable to the detection of zinc, lead and cadmium by the biosynthesis of green fluorescent protein (GFP) accumulating inside the cells. Flow cytometry was used to investigate the fluorescence induced by the MTEs. A curvilinear response to zinc between 0 and 25 mg/L and another curvilinear response to cadmium between 0 and 1.5 mg/L were highlighted in liquid media, while lead did not produce exploitable results. The response relating to a Zn^2+^/Cd^2+^ ratio of 10 was further investigated. In these conditions, *E. coli* pP_ZntA_gfp responded to cadmium only. Several contaminated soils with a Zn^2+^/Cd^2+^ ratio of 10 were analyzed with the biosensor, and the metallic concentrations were also measured by atomic absorption spectroscopy. Our results showed that *E. coli* pP_ZntA_gfp could be used as a monitoring tool for contaminated soils being processed.

## 1. Introduction

Metallic trace elements (MTEs) can be found in the Earth’s crust at relatively low concentrations (less than 0.1%) [1]. Understanding their relationships with living beings (animals, men, plants, bacteria) is still a current topic in numerous fields, such as environmental protection, human health and agriculture productivity [2]. Some of those MTEs are toxic at high concentrations, although some of them can have metabolic key functions at weaker concentrations [3]. So far, no metabolic role could be highlighted for cadmium, lead and mercury, and those three elements are considered as the most problematic as regards environmental problems [3,4,5]. On the other hand, some other elements, such as zinc, copper, manganese and metalloids (e.g., selenium), are needed in agricultural lands, having a positive impact on agricultural productivity and human health [2]. Cadmium (Cd), mainly used in batteries, pigments, metal coatings and plastics, is known as a human carcinogen, also causing kidney, lung and bone diseases [2]. Fertilizers and atmospheric depositions have considerably increased the global Cd concentration in soils (naturally present at 0.1–1 mg/kg [6]). This element can also be found in natural minerals (greenockite (CdS), otavite (CdCO_3_), *etc.*), but this source is quite limited [6]. Lead (Pb) reaches a mean concentration of 14.8 mg/kg in the continental crust and is usually found in soils at concentrations between 10 and 67 mg/kg [5,7]. It also results from many human activities (fossil fuel burning, mining, manufacturing, batteries, pipes, *etc.*) and is highly toxic. It can cause damages to brain and kidneys and is very harmful to pregnant women, leading to miscarriage [8]. It tends to accumulate in the environment, mainly in soils and sediments [7]. Zinc (Zn), less toxic than Pb and Cd, is typically found in soils at 10–100 mg/kg. It is an essential micronutrient for biota at low concentrations, although higher quantities cause toxic effects to plants, soil-dwelling organisms and microorganisms. It is considered that Zn is not dangerous to humans, and its potential negative effects are rather observed on soil biota and soil functioning [9].

The determination of the effect of heavy metals on the environment and health has been the subject of many studies. MTEs are widespread in the environment and can interact with the soil and living organisms through many pathways. One single MTE can be found in many forms; this is the speciation concept. Soil is a complex matrix composed of solid, liquid and gaseous phases, and the MTEs are spread in those different zones and in their different forms. The elements can be found free in solution or combined with solid materials by adsorption or absorption. They can also form complexes or precipitates [10]. Their speciation depends on many parameters: the element nature, its electronegativity, its charge, the presence or the absence of living organisms, the presence of ligands, temperature, moisture, pressure, pH, redox potential, cationic exchange capacity, *etc.* [11] Consequently, dealing with the environmental and health problems related to MTEs requires taking this complexity into account. For these reasons, quantitative data about the total and extractable MTE concentrations are usually required to understand their effects.

The treatment of soils contaminated with MTEs can be undertaken by different techniques: non-biological methods (isolation, electro-kinetic remediation, soil leaching, adsorption, heat treatment, physical solidification, chemical improvers, chemical curing lamp remediation, washing and compounding) and biological methods (phytoremediation, animal remediation, microbial remediation) [8]. In some cases, it is necessary to assess the efficiency of the process during the treatment period, especially for the techniques that involve a progressive decontamination. This follow-up can be processed by measuring the concentrations of the MTEs considered in the process. For some techniques, especially those that use microorganisms, the determination of the bioavailable concentration of the MTEs can be very helpful. The assessment of toxicity can provide useful data, as well. In this regard, biosensors are powerful tools. They are defined as analytical devices integrating a biological recognition element with a physical transducer able to generate a measurable signal proportional to the concentrations of the analytes [12]. Many genera and species were used as biosensors in previous studies, such as specific strains of *Clostridium butyricum*, *Photobacterium phosphoreum*, *Escherichia coli*, *Pseudomonas fluorescens*, *Pseudomonas putida*, *Staphylococcus aureus*, *Alcaligenes eutrophus* and *Synechococcus* [13,14,15,16,17,18,19,20,21,22,23,24,25,26,27,28]. Among the biosensors, some are based on genetically-engineered systems involving a reporter gene inserted in an artificial plasmid and coding the sequence of a molecule emitting a light signal, such as the “green fluorescent protein” (GFP) extracted from the jellyfish *Aequorea victoria* [29].

Flow cytometry has been used in this work to assess relative fluorescence values directly, since this method allows quantifying fluorescence intensities at the single-cell level. In this way, the values measured are directly proportional to the number of biosensors present in the samples, and artifacts can be avoided (*i.e*., fluorescent soil particles).

Here, we studied the strain *Escherichia coli* pP_ZntA_gfp and its response to three metals: Zn, Pb and Cd. The aim was to determine the effect of those three MTEs on the synthesis of GFP. Flow cytometry was used as the analysis tool. A calibration was achieved in liquid media before assessing the ability of the strain to provide information about the bioavailable concentrations of MTEs in samples of contaminated soils. The results were compared to the pseudo-total and extractable MTE concentrations determined by atomic absorption spectrometry.

## 2. Experimental Section

### 2.1. Strain

*Escherichia coli* strain K-12 MG1655 was used in all of our experiments. We selected this microorganism, because it is fully sequenced, well known, and its metallome and its homeostasis relating to the MTEs were studied previously [30]. Moreover, many biosensors were engineered from that strain, able to detect MTEs in the environment, but also microorganisms that can precipitate Zn and Cd under the form of sulfides [31]. Finally, this strain is not pathogenic. The reporter gene, inserted in a plasmid construction, corresponded to GFP, activated by the promoter pP_ZntA_gfp. ZntA is a P_1B-2_-ATPase, which consumes ATP to export MTEs against their gradient. This ATPase is regulated at the transcriptional level by ZntR (regulatory protein). If ZntR is linked to zinc, cadmium, lead or mercury and to DNA (in a region close to the promoter of ZntA), it activates the transcription of ZntA and the exportation of these metallic elements. Without one of these bindings, ZntR is hydrolysed by proteases, and the ZntA gene is inhibited [32,33,34,35,36,37]. The activation of the promoter of ZntA appears especially at high Zn, Cd, Pb or Hg concentrations, which means that this promoter can be used to assess the availability of these elements specifically. The genetically-engineered strain was provided by the Weizmann Institute of Science (234 Herzl St., Rehovot 7610001, Israel). The original sample was first kept at −80 °C. The working seeds were prepared as follows: 100 µL of the original sample were added to 100 mL of sterile LB (lysogeny broth) medium. The bacterium was cultivated for 18 h (37 °C, 120 rpm). Then, 30 mL of culture were mixed with 20 mL of sterile glycerol. After homogenization, the suspension was introduced into 1.5-mL sterile tubes, which were conserved at −80 °C.

### 2.2. Soil Samples

Three samples were tried and were obtained from the nature reserve of Sclaigneaux (Wallonia, Belgium). This site was monitored by the faculty of Gembloux Agro-Bio Tech (University of Liège) and was contaminated with Cd, Pb and Zn by an atmospheric deposition mainly stemming from a former Zn-Pb ore treatment plant [38]. Two types of samples were tested in the biosensor experiments. For each sample, the soil was sieved (maximal particle size: 2 mm) or finely ground (maximal particle size: 200 µm). A stove was used to dry the soil samples before the sieving step.

### 2.3. Cell Culture

All products were provided by Sigma-Aldrich. The strain was routinely grown in LB medium (10 g/L tryptone, 5 g/L yeast extract, 5 g/L NaCl) at 37 °C. A primary culture was prepared in 100 mL LB medium inoculated by 50 µL of a working seed. After incubation for 18 h (37 °C, 120 rpm), 500 µL of culture were added to 2.5 mL LB medium with or without Zn addition, leading to a resulting Zn concentration of 10.325 or 0.325 mg/L, respectively. These primary cultures were used to make an exploratory investigation of the effect of Zn^2+^ on fluorescence (see Section 3.1.1).

Several assays required a low-nutrient medium and were performed using a modified glycerol-glycerophosphate medium (GGM), adapted from [39], containing, per liter, 5 g glucose, 8.4 g MOPS (3-(N-morpholino)propanesulfonic acid), 1 g KCl, 0.87 g K_2_SO_4_, 0.1 g MgCl_2_, 1 g disodium β-glycerophosphate, 1 g NH_4_Cl, 0.01 g CaCl_2_, 0.109 mg CoCl_2_∙H_2_O, 0.018 mg CuCl_2_, 0.06 mg H_3_BO_3_, 0.064 mg MnCl_2_, 0.04 mg Na_2_MoO_4_∙2H_2_O, 0.02 mg NiCl_2_∙6H_2_O [39]. Culture media were supplemented with 25 µg/mL kanamycin, as suggested by the strain provider. Metal-supplemented media were obtained by the addition of filtered metallic solutions prepared from Titrisol^®^ solutions (Merck, Darmstadt, Germany) of ZnCl_2_, CdCl_2_ and Pb(NO_3_)_2_. Primary cultures were inoculated with 250 µL or 50 µL of vortexed working seeds, for 100 mL of minimum medium or LB, respectively, and incubated for 20 h (37 °C, 120 rpm). All biosensor assays were performed in 12 well-plates, each well containing 2.5 mL culture medium (supplemented with MTEs when needed) inoculated by 0.5 mL of primary culture. The soil analyses were performed on soil samples, previously dried and sieved at 2 mm or ground at 0.2 mm, next incorporated into the low-nutrient culture medium. To ensure a response within the biosensor range, three concentrations were tested for each soil: 0.025 g/mL, 0.05 g/mL and 0.1 g/mL.

### 2.4. Propidium Iodide Staining

Propidium iodide was used to assess the cell viability in the presence of Cd, Pb and Zn. This molecule can penetrate into the cells, the membranes of which have been damaged. Then, it binds to the bases of DNA. In these conditions, fluorescence can be measured with an excitation wavelength of 540 nm. The staining protocol was as follows: a stock solution of propidium iodide was prepared (1 g/L), and 10 µL of staining solution were added to 1 mL of each cell sample. The resulting suspension was left to rest for 20 min at 37 °C before being centrifuged. The supernatant was removed, and 1 mL of fresh medium was added to the pellet (see Section 2.3). The samples were vortexed before being centrifuged one more time, and the supernatant was removed as before. The pellet was re-suspended in 1 mL of fresh medium, and the resulting suspension was conserved at 4 °C before being analyzed by flow cytometry. A control was prepared by heating a cell suspension at 65 °C for 30 min. The resulting suspension was next stained as described before and considered as a reference of non-viable cells.

### 2.5. Recovery of Biosensors from Soils by Nycodenz^®^

Prior to flow cytometry analyses, the biosensors grown in soil-containing samples were separated from soil particles and recovered by a density gradient protocol. Nycodenz^®^ (provided by Progen Biotechnik GmbH Maaßstraße 30, 69123 Heidelberg, Germany), a highly soluble, non-ionic and non-toxic agent, has been used for this purpose for several years [40] and can be used in fluorescence-based assays [41]. Stock solutions were prepared by the addition of 8 g Nycodenz^®^ with 10 mL sterile distilled water, leading to a density of 1.3 g/mL, following a protocol described by [42]. To collect the biosensors, 1 mL of this solution was slowly added to 1 mL of homogenized cell culture in 2-mL Eppendorf tubes and centrifuged 5 min at 18,000 g. After centrifugation, 250 µL were sampled at the Nycodenz^®^-culture media interface where the biosensor formed a visible halo. Those samples were further analyzed by flow cytometry.

### 2.6. Flow Cytometry Analysis

Flow cytometry analyses were performed on an Accuri C6 Flow Cytometer (Accuri cytometers, Inc., Ann Arbor, MI, USA). The cell suspensions were sampled for 40 s, at medium speed for the LB medium and at high speed for the low nutrient medium. Data were further treated using the software CFlow v 1.0 provided with the cytometer.

### 2.7. Metal Analysis by Atomic Absorption Spectroscopy

Zinc, Pb and Cd were analyzed by atomic absorption spectroscopy (AAS). For each soil sample, three different extraction conditions were used to determine the total, available (fraction presenting long-term mobility) and soluble concentrations (protocol described by Liénard *et al.* [43]). Prior to the analyses, the soil samples were ground and sieved at 2 mm. As for the extraction of the soluble fraction, 5 g of dried soil were extracted by 50 mL of 0.01 M CaCl_2_ solution during two hours under agitation. The solutions were then filtered (Whatman^®^ Grade 595 1/2) and analyzed after less than 24 h. The available fraction was determined by extracting 10 g of dried soil by 50 mL of an extractive solution containing EDTA (0.002 M), ammonium acetate (0.5 N) and acetic acid (0.5 N). It is considered that EDTA can be used to estimate the long-term mobility of the metallic elements [44]. The solution was next agitated for 30 min, filtered as previously and analyzed after less than 24 h. The determination of pseudo-total MTE (digestion with aqua regia) concentrations was made as follows: 3 g of soil sample were treated by a mixture of 22.2 mL HCl 37% and 7.5 mL HNO_3_ 65% (aqua regia) in a Gerhardt asher. The resulting solution and the nitric acid 0.5 M from the vapor trap were then pooled in a 100-mL vial, completed with distilled water. The solution was then filtered (Whatman^®^ Grade 602 H 1/2) and analyzed after less than 24 h. The quantification of Zn, Cd and Pb was then performed using a SpectrAA 110/220, VARIAN AAS (flame atomic absorption spectrometry). The detection limits for aqua regia/available metals for Cd, Pb and Zn were, respectively, 0.66–0.10 mg/kg, 3.33–0.5 mg/kg and 0.33–0.05 mg/kg (Zn). As part of the quality control program for the study, a standard reference material was used and analyzed with each set of samples. Those experiments were undertaken by the lab of the “Axe Echanges Eau-Sol-Plante” of the BIOSE (“Biosystem Engineering”) department (Gembloux Agro-Bio Tech, Passage des Déportés 2, 5030 Gembloux, University of Liège, Belgium).

## 3. Results and Discussion

### 3.1. Development of Fluorescence-Based Biosensors for MTE Detection in Liquid Media

#### 3.1.1. Effect of Zn Concentration and Incubation Time

**Figure 1:**
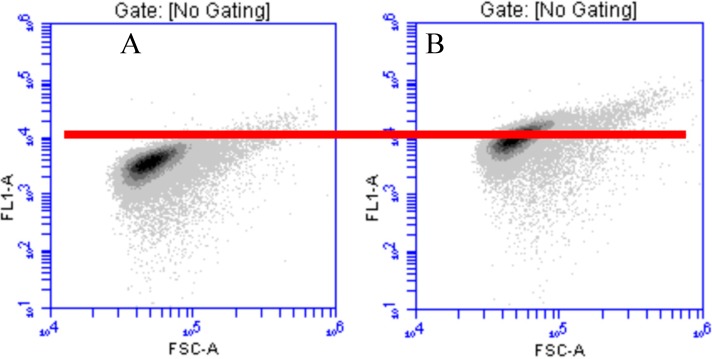
Cytogram (fluorescence *versus* side scatter in arbitrary units) of *E. coli* pP_ZntA_gfp grown for 22 h in LB medium containing 0.325 mg/L Zn^2+^ (**A**) and 10.325 mg/L Zn^2+^ (**B**). The red line indicates a fluorescence of 10^4^ A.U. related to the basic level of GFP expression.

The first experiments aimed to assess the effect of Zn concentration on the synthesis of GFP by the strain *Escherichia coli* pP_ZntA_gfp. Flow cytometry was used on the primary cultures containing Zn^2+^ concentrations of 0.325 or 10.325 mg/L (see Section 2.3) after 4 h of incubation. The cytograms (Figure 1) clearly showed the effect of Zn^2+^ on the fluorescence induction with a shift of the similar scatter from an average fluorescence of 4000 to an average of 12,000 A.U. when the Zn^2+^ concentration increased from 0.325 to 10.325 mg/L.

**Figure 2:**
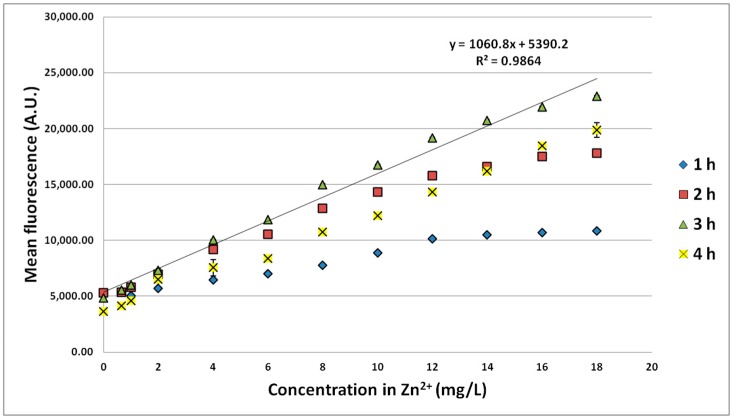
Fluorescence of the strain *E. coli* pP_ZntA_gfp induced by different Zn^2+^ concentrations and for different incubation times. Confidence intervals are indicated (95%).

**Figure 3:**
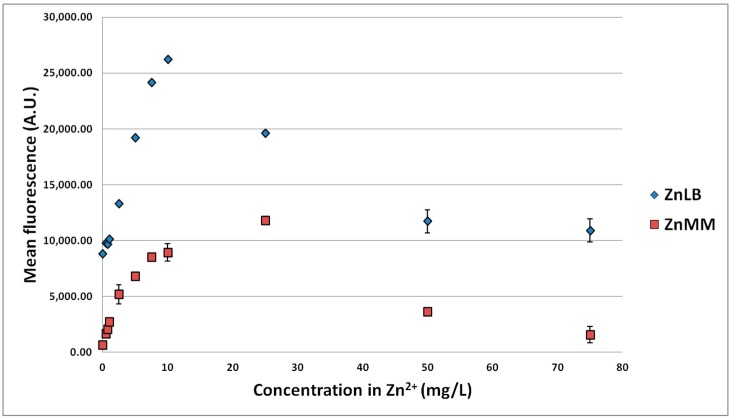
Fluorescence of the strain *E. coli* pP_ZntA_gfp induced by different Zn^2+^ concentrations in low-nutrient (ZnMM, Minimal Medium) and LB (ZnLB, Lysogeny Broth) culture media. Confidence intervals are indicated (95%).

**Figure 4:**
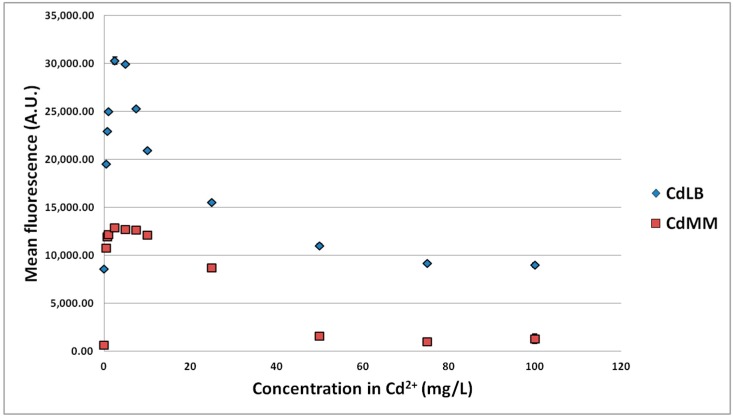
Fluorescence of the strain *E. coli* pP_ZntA_gfp induced by different Cd^2+^ concentrations in low-nutrient (CdMM) and LB (CdLB) culture media. Confidence intervals are indicated (95%).

**Figure 5:**
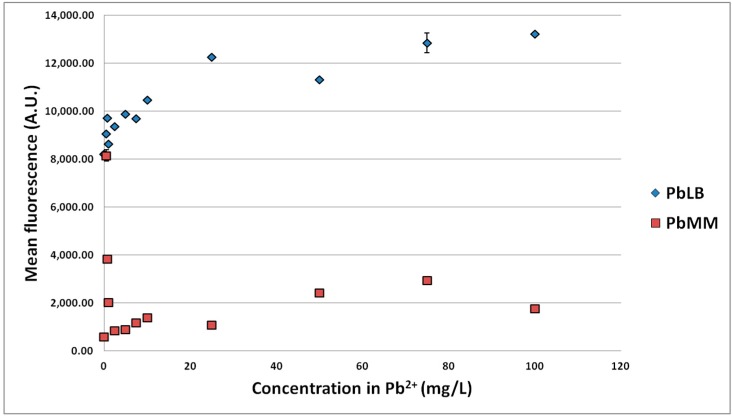
Fluorescence of the strain *E. coli* pP_ZntA_gfp induced by different Pb^2+^ concentrations in low-nutrient (PbMM) and LB (PbLB) culture media. Confidence intervals are indicated (95%).

Different Zn^2+^ concentrations were also tested up to 18 mg/L, and fluorescence was measured after 1 to 4 h of incubation in order to determine the optimal conditions for using the biosensor. A basic fluorescence of about 5000 A.U. was recorded for each incubation time, except after 4 h, with a value of about 4000 A.U. (Figure 2). At least 6000 events were analyzed to calculate the confidence intervals in Figure 2, Figure 3, Figure 4 and Figure 5.

For each concentration, the maximal fluorescence was detected after 3 h of incubation. The decrease of fluorescence observed after 4 h could result from the lack of nutrients. The growth may be affected by the consumption of the nutrients of the broth medium, which could induce mortality events and, therefore, have an impact on the measurements. In the conditions we have applied, a sufficient linearity (correlation coefficient R^2^ = 0.986) was recorded up to 18 mg/L Zn^2+^ and expressed by the equation: fluorescence [A.U.] = 1060.8 [Zn^2+^] + 5390.2. A higher R^2^ of 0.997 was reached in the working range 0–10 mg/L.

#### 3.1.2. Effect of MTE Concentrations

Higher concentrations of Zn^2+^ (up to 75 mg/L) along with Cd^2+^ and Pb^2+^ (up to 100 mg/L) were further tested in LB medium and also in the low-nutrient medium (3 h of incubation in this medium containing different MTE concentrations after 18 h of culture in the basic LB medium). The resulting fluorescence was measured by flow cytometry.

These experiments (Figure 3, Figure 4 and Figure 5) confirmed the previous results concerning the signal of the biosensor to Zn^2+^ in the working range 0–10 mg/L. Over the larger range with the 3 MTEs, the LB medium globally led to a higher signal than the low-nutrient medium and Pb to a lower signal than both Zn and Cd. Moreover, the profile *versus* MTE concentrations reveals a maximum signal. The higher fluorescence induction in LB medium can be explained by the presence of yeast extract and proteins in the culture medium, boosting cell growth and GFP biosynthesis, leading to a 10-fold higher fluorescence without MTEs. The maximal induction for Zn was observed between 10 and 25 mg/L for the LB medium and at about 25 mg/L for the low-nutrient medium. It was observed at about 2.5 mg/L in both media for Cd and at 100 or 0.5 mg/L for Pb in LB and low-nutrient medium, respectively. It is interesting to note that the maximal induction observed with Cd is superior to the one with Zn. This was already reported by Binet and Poole [45] with a genetically-engineered plasmid P_ZntA_lacZ in *E. coli*. Gireesh-Babu and Chaudhari [46] confirmed our results (Section 3.1.1) regarding the variation of the biosensor signal *versus* incubation time. However, they reported a decrease of the signal with the increase of incubation time, probably because of more complexing reactions [46].

Since the signal measured with Pb was quite chaotic and lower than the ones for Cd and Zn, it was relatively difficult to model it. Consequently, the biosensor efficiency was revealed to be relatively low and not applicable to Pb. A significant difference of fluorescence was observed between the different metals. Among the potential explanations, toxicity could play a role. While an excess of both organic and inorganic Pb compounds is toxic due to the potential formation of many derivatives, the organic compounds are generally considered as more toxic and stable than Pb itself [47]. Indeed, Pb is involved in many biochemical reactions, which can affect the cell metabolism. It can: (i) form mercaptides with cysteine in enzymes and more generally in proteins; (ii) inhibit most enzymes containing one functional –SH group; (iii) affect some ATPases and cellular oxidation processes; or (iv) alter nucleotides and nucleic acids. All of these reactions could explain a decrease of cell growth and some dysfunctions in the biosensor. The affinity of the biosensor for Pb and its availability in solution could also be lower than for Zn and Cd. The most plausible explanation is the lower availability, since Pb^2+^ may form some insoluble precipitates (e.g., PbS) or many stable complexes with both soft and hard donor atom ligands [48].

The response of the biosensor to Cd cannot be modelled by a simple linear regression as in the range 0–10 mg/L for Zn. Indeed, correlation coefficients of 0.963 and 0.944 were obtained for the results in the LB medium up to 1 mg/L Cd and in the low-nutrient medium up to 0.75 mg/L Cd, respectively. However, these linear regressions considered a low number of signal values. Therefore, a different approach must be undertaken.

Cadmium, Pb and Zn are transported by different systems in the cells, and those transport systems are identical for the three metallic elements in *E. coli*. A modification of the metallic concentration in the medium causes stress to the cells, leading to a modification of the expression of some genes and, as a consequence, an alteration of protein synthesis (e.g., enzymes) [49]. The promoter located on the plasmid pP_ZntA_gfp is activated by the action of an enzyme that could be exploited for biodetection. Therefore, the model developed by Michaelis–Menten can be considered [50]. This model is based on the formation of the substrate-enzyme complex to characterize enzymatic reactions. Here, we adapted that model to characterize and to describe the relationship between fluorescence and the metallic concentration.

The effect induced by Zn^2+^ and Cd^2+^ was also investigated by propidium iodide. Our results showed that the cell viability had not been modified by Zn^2+^ and Cd^2+^ concentrations between 0 and 25 mg/L. In these conditions, the red fluorescence was always inferior to 1000 A.U., which means that there was no negative effect on the cell membranes.

#### 3.1.3. Impact of Zn^2+^/Cd^2+^ Ratio on Biosensor Response

**Figure 6:**
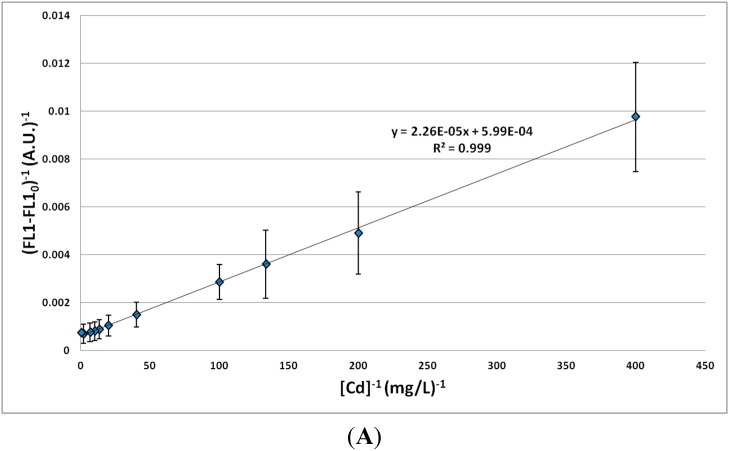

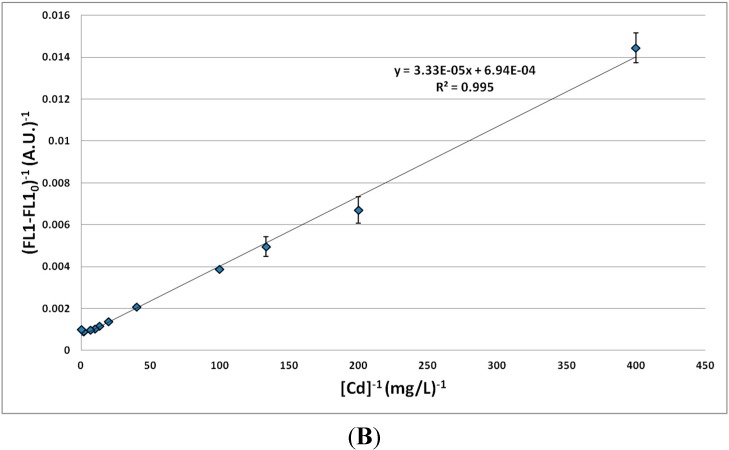
Linear regression between fluorescence and Cd^2+^ concentration (Zn^2+^/Cd^2+^ ratio = 10) based on different working seeds (**A**) or one single working seed (**B**). Confidence intervals are based on four replicates.

Since the signal profiles *versus* MTE concentrations were similar for Zn and Cd in the low-nutrient medium, the linear regression was studied more deeply with the combination of Zn and Cd. Lead was not considered here for the reasons mentioned in Section 3.1.2. Two different Zn^2+^/Cd^2+^ ratios were tested. A ratio of 50 induced a response significantly similar to the one corresponding to the sole Zn element. On the contrary, a ratio of 10 induced a response significantly similar to the one of Cd alone. This ratio was further investigated. The formula of the model of Michaelis–Menten, adapted to this work, is given by Equation 1.

(1) $$\frac{1}{FL1-FL1_{0}}\;=\;\frac{1}{\left[MTE\right]}*\;\frac{K_{m}}{FL1_{max}\;-\;FL1_{0}}+\;\frac{1}{FL1_{max}\;-\;FL1_{0}}$$

where FL1 is the mean fluorescence, FL1_0_ is the initial fluorescence without MTEs, [MTE] is the concentration of MTEs, K_m_ is the Michaelis constant and FL1_max_ is the maximal value of fluorescence. This equation was investigated on the basis of the mean fluorescence values according to the concentrations of MTEs, with a Zn^2+^/Cd^2+^ ratio of 10 (Figure 6).

The correlation coefficients higher than 0.99 confirmed the relevance of this approach for MTE concentrations ranging between 0.0025 and 2.5 mg/L for Cd^2+^ and between 0.025 and 25 mg/L for Zn^2+^. When Zn is considered alone, a correlation coefficient of 0.999 may be reached, which is much better than for the simple linear regression. A higher variability (larger confidence intervals) was observed when data from different working seeds were considered. This result suggests that a calibration should be carried out with each working seed. In these conditions, the method is reliable and reproducible. As an example, for Cd^2+^, one specific working seed (four repetitions) led to a K_m_ value of 0.0378 ± 0.0046 mg/L, a mean FL1_0_ value of 682 ± 14 A.U. and a mean FL1_max_ value of 1249 ± 44 A.U., whereas a larger variability was observed between the different working seeds, with a mean K_m_ value of 0.0467 ± 0.0386 mg/L, a mean FL1_0_ value of 637 ± 44 A.U. and a mean FL1_max_ value of 2856 ± 3203 A.U.

### 3.2. Soil Analysis

The method developed in the former sections was used to assess the ability of *E. coli* pP_ZntA_gfp to provide information about the bioavailable MTEs concentrations of soil samples. The biosensor results were compared to analytical data obtained by atomic absorption spectroscopy.

#### 3.2.1. Soil Characterization by Atomic Absorption Spectroscopy

Three samples from a soil contaminated by Zn, Pb and Cd were analyzed by atomic absorption spectroscopy (AAS) after three different extraction procedures. The total concentrations of Cd, Pb and Zn (Table 1) lie within the average ranges for contaminated soils [51], while Soil 3 (S3) was slightly less contaminated.

**Table 1:** Characterization of Cd, Pb and Zn concentrations in 3 samples from a contaminated soil by atomic absorption spectroscopy following different extraction protocols. MTE, metallic trace element.

Soil ID	Soil pH	Extraction Method								
		Aqua Regia *Total MTEs* (mg/kg)			EDTA + Ammonium Acetate Solution, pH 4.65 *Available MTEs* (mg/kg)			CaCl_2_ Solution *Soluble MTEs* (mg/kg)		
		Cd	Pb	Zn	Cd	Pb	Zn	Cd	Pb	Zn
S1	6.9	5	131	410	3	73	69	ND *	ND *	0.547
S2	6.8	4	117	318	3	69	91	ND *	ND *	0.274
S3	6.9	2	59	200	1	30	51	ND *	ND *	0.434

* Quantity below the limit of detection (LOD) of the system.

Contaminated soils usually contain 100 to 1000 mg/kg of Zn and 1 to 10 mg/kg of Cd, whereas uncontaminated soils naturally contain 10 to 100 mg/kg of Zn and 0.1 to 1 mg/kg of Cd [6,9]. However, the contaminations with these metals may exceptionally reach extreme values of 50,000 mg/kg and 1000 mg/kg, respectively [51]. The average Zn^2+^/Cd^2+^ ratio of the samples S1 to S3 were around 100, 30 and 10 regarding the total, the environmentally available and the soluble concentrations, respectively. By comparison, the average ratio in the terrestrial crust (rocks and areas) is about 200, due to a higher concentration of Zn [51]. Moreover, Cd shows a higher long-term mobility than Zn in soils, explaining the decrease of the ratio when using less strong extraction techniques.

#### 3.2.2. Soil Characterization by Flow Cytometry and MTEs Bioavailability Assessment

Due to strong variations observed between the working seeds, calibrations were repeated for each of them. A Zn^2+^/Cd^2+^ ratio of 10 was also used for these calibrations, since it was close to the ratios observed for soluble contents of MTEs in the soil samples. After the culture in soil-containing media and the recovery of bacteria by the density gradient technique, the biosensors were analyzed by flow cytometry (four replicates per sample). In order to ensure robust data, the samples with less than 6000 events in 40 s were discarded. While a count of 25,000 events was easily reached for calibration samples, some soil samples did not reach the threshold of 6000 events. Soil particles were suspected to carry biosensors along during the centrifugation step. The samples presenting a standard deviation of fluorescence higher than 300% were also discarded. Such high variations were probably caused by the presence of biosensor aggregates and should thus not be taken into account. As expected from an increase of extractible material and extraction surface, fluorescence increased along with the soil concentration and with grinding.

The bioavailable concentrations of Cd^2+^ were calculated on the basis of the equations presented before (see Section 3.1.3). The results for Cd^2+^ are summarized in Table 2. Here, Cd^2+^ was considered alone, because it is not possible to quantify both ions at the same time. As was mentioned before (see Section 3.1.3), a Zn^2+^/Cd^2+^ ratio of 10 allows detecting Cd^2+^ alone. However, a higher ratio (at least 50) allows detecting Zn^2+^ exclusively.

**Table 2:** Concentrations of bioavailable Cd^2+^ in the studied samples according to the soil concentrations and the particle size. A mass of 25, 50 or 100 mg of soil was added to 1 mL of broth medium (Column 2), and the concentration of MTEs in soil (mg/kg) was deduced from fluorescence measurements (Columns 3, 4, 5, 6).

Soil ID	Soil Concentration (mg/mL)	Bioavailable Cd^2+^ in Soils (mg/kg)			
		Soil Sieved at 2 mm		Soil Ground at 0.2 mm	
		Average	Amplitude	Average	Amplitude
S1	25	0.26	0.14–0.38	0.31	0.30–0.39
	50	0.22	0.11–0.36	0.20	0.17–0.50
	100	0.20	0.06–0.41	0.15	0.13–0.50
S2	25	0.33	0.16–0.50	0.34	0.32–0.36
	50	0.24	0.14–0.41	0.22	0.16–0.27
	100	0.14	0.10–0.23	0.14	0.13–0.24
S3	25	0.14	0.06–0.22	0.22	0.21–0.22
	50	0.10	0.04–0.13	0.13	0.10–0.13
	100	0.08	0.03–0.10	0.08	0.07–0.10

A decrease of metallic bioavailability was observed with an increasing soil concentration. This suggests that stirring was not efficient enough to prevent a part of the sample from remaining unstirred at the bottom of the well and thus to be protected from exchanges with the liquid phase. Therefore, the rotating shaker should be replaced by another means in order to improve mixing and solid/liquid extraction performances. Regarding the results that will be further discussed at a soil concentration of 25 mg/mL, the bioavailability values reached for 2-mm sieved soils or 0.2-mm ground soils were significantly similar; a linear regression was applied to those results and led to an R^2^ coefficient of 0.8 with a *p*-value inferior to 0.01 for the slope. Consequently, it can be assumed that there is no significant difference resulting from particle size on Cd determination. However, our results did not focus on Zn, because the Zn^2+^/Cd^2+^ ratio was close to 10.

The bioavailable Cd^2+^ concentrations estimated by the biosensor were 0.26, 0.33 and 0.14 mg/kg of soil for the samples S1, S2 and S3, respectively (sieved at 2 mm). The metal bioavailabilities of the two first soils were relatively close, while the third was lower. These results are consistent with the AAS results, which showed lower MTEs concentrations for S3 according to the different extraction methods. The estimated bioavailable concentrations are comprised between the concentrations obtained with EDTA and CaCl_2_ extraction protocols, respectively corresponding to the available metal fraction (or potentially hazardous fraction) and the soluble fraction. The metal fraction available for microorganisms seems then to be higher than the easily recoverable fraction, estimated through the CaCl_2_ extraction. However, the AAS results for this last extraction method should be considered carefully, because the values are inferior to the limit of detection of the method.

#### 3.2.3 Relevance of Biosensors for Soil Characterization

*E. coli* pP_ZntA_gfp showed an interesting potential to be used as a biosensor for Cd or Zn availability in soils. Indeed, its detection limits are in line with those of the most common chemical analysis techniques (see Table 3). This method also presents a good repeatability and reproducibility, if the initial state of the population is taken into account in a calibration procedure for each working seed. Protein expression in bacteria is indeed subjected to variations according to extrinsic and intrinsic sources of noise. A better understanding and control of these phenomena would enable one to avoid the systematic calibration step for each seed and, as a consequence, would facilitate the use for routine analyses.

The sensitivity of the method can be assessed on the basis of the K_m_ values. Here, we focused our study on the case of Cd^2+^, more toxic and harmful than Zn^2+^. In our case, the K_m_ value relating to Cd^2+^ was calculated (0.0467 mg/L), which corresponds to a soil concentration of 0.467 mg/kg of soil. This value means that a concentration of 0.0467 mg/L corresponds to the half of maximal induction, taking account of the basic fluorescence. The reference value for cadmium concentration in Walloon soils is cited in the decree of the Walloon Government for soil management [52]. The value of 0.2 mg/kg is applicable to natural, agricultural, residential, commercial and industrial soils [52]. The limit and intervention values are higher and consequently measurable by the present technique, although a soil dilution may be necessary. The toxicities of Cd^2+^ and Zn^2+^ in *E. coli* are other important parameters to take into account to assess the applicability of the technique to contaminated samples. The minimal inhibitory concentrations of Zn^2+^ and Cd^2+^ in *E. coli* are 65 and 56 mg/L, respectively [53]. Such concentrations may be measured by the present technique after having applied the right dilution to the soil considered.

Both sensitivity and dynamic range relating to Cd^2+^ and Zn^2+^ should be improved, although the technique was not deeply characterized for Zn^2+^ as in the case of Cd^2+^. For instance, it is possible to add a self-activation loop on the same [46,54,55] or on different plasmids [56,57]. This technique allows reaching a better sensitivity. Another point is that microbial bioavailability observed in laboratory or *in situ* conditions might differ. Indeed, in the soil, microorganisms often form biofilms on solid surfaces. This increased proximity, possibly associated with physiological shifts and potential interspecific interactions, might increase the environmental bioavailability.

**Table 3:** Limits of detection and ranges of the most used cadmium determination methods [58,59,60].

Method	Abbr.	Limits of Detection and Dynamic Range												
		1 ppq		1 ppt		1 ppb				1 ppm		10^3^ ppm		
X-ray fluorescence spectrometry	XRF													
Inductively coupled plasma-mass spectrometry	ICP-MS													
Inductively coupled plasma-atomic emission spectroscopy	ICP-AES													
Graphite furnace-atomic absorption spectroscopy	GFAAS													
Atomic absorption spectroscopy	AAS													
*E. coli* pP_ZntA_gfpl														

Dark grey: solid and liquid samples; light grey: liquid samples only. (ppm = part per million, ppb = part per billion, ppt = part per trillion, ppq = part per quadrillion).

## 4. Conclusions

This study highlights the potential of biosensors for the characterization of metallic soil contaminations. Indeed, we have shown that the bioengineered strain *E. coli* pP_ZntA_gfp can be used as a tool to estimate the bioavailability of Cd in soils. The applicability to Zn should be further investigated. The responses observed for Pb were less clear and depended on various physiological factors. This metal is therefore more problematic to monitor with this biosensor. The method that was developed to analyze Zn^2+^ and Cd^2+^ showed a very good linearity and repeatability, although a calibration was necessary for each working seed. The detection limits of this method are close to those of most chemical methods.

Our biosensor is a living organism comparable with those that are used in bioremediation processes. Biosensors give information about the chemical species of MTEs available to the micro-organisms. This bioavailable fraction, which is targeted in bioremediation processes, does not correspond to the fraction determined by the different extraction methods used in this study. These results highlight the need for developing tools to measure the bioavailable fraction specifically. Bio-detection depends on metabolic activities comparable with the one developed by the bacteria exploited in such treatments. Therefore, chemical and biochemical techniques can be considered as complementary. Here, we have showed that *E. coli* pP_ZntA_gfpl could be a suitable biosensor for soil contamination monitoring and for the determination of bioremediation relevance. However, it is important to understand that the use of our procedure alone cannot provide information about the nature of the metallic elements that are present in the samples. Another important conclusion is that it is not possible to measure Cd^2+^ and Zn^2+^ simultaneously, because the Zn^2+^/Cd^2+^ ratio determines what ion can be measured through our technique: Zn^2+^ (Zn^2+^/Cd^2+^ ratio superior to 50) or Cd^2+^ (Zn^2+^/Cd^2+^ ratio inferior to 10).
